# A Structural Entropy Measurement Principle of Propositional Formulas in Conjunctive Normal Form

**DOI:** 10.3390/e23030303

**Published:** 2021-03-04

**Authors:** Zaijun Zhang, Daoyun Xu, Jincheng Zhou

**Affiliations:** 1 College of Computer Science and Technology, Guizhou University, Guiyang 550025, China; zjzhang1987@outlook.com; 2 Key Laboratory of Complex Systems and Intelligent Computing and School of Mathematics and Statistics, Qiannan Normal University for Nationalities, Duyun 558000, China; guideaaa@126.com

**Keywords:** SAT problem, the structural complexity, structural entropy, CNF formula, structural properties

## Abstract

The satisfiability (SAT) problem is a core problem in computer science. Existing studies have shown that most industrial SAT instances can be effectively solved by modern SAT solvers while random SAT instances cannot. It is believed that the structural characteristics of different SAT formula classes are the reasons behind this difference. In this paper, we study the structural properties of propositional formulas in conjunctive normal form (CNF) by the principle of structural entropy of formulas. First, we used structural entropy to measure the complex structure of a formula and found that the difficulty solving the formula is related to the structural entropy of the formula. The smaller the compressing information of a formula, the more difficult it is to solve the formula. Secondly, we proposed a λ-approximation strategy to approximate the structural entropy of large formulas. The experimental results showed that the proposed strategy can effectively approximate the structural entropy of the original formula and that the approximation ratio is more than 92%. Finally, we analyzed the structural properties of a formula in the solution process and found that a local search solver tends to select variables in different communities to perform the next round of searches during a search and that the structural entropy of a variable affects the probability of the variable being flipped. By using these conclusions, we also proposed an initial candidate solution generation strategy for a local search for SAT, and the experimental results showed that this strategy effectively improves the performance of the solvers CCAsat and Sparrow2011 when incorporated into these two solvers.

## 1. Introduction

Given a propositional formula in conjunctive normal form with variables $\left\{x_{1},x_{2},\cdots ,x_{n}\right\}$ and clauses $\left\{c_{1},c_{2},\cdots ,c_{m}\right\}$, where $c_{i}$ is the disjunction of some literals, a literal refers to the variable *x* or its negation ¬x. The satisfiability problem consists of finding an assignment for the variables so that all clauses are satisfied. The satisfiability (SAT) problem is a core problem in computer science, and many real-world applications, such as hardware and software verification, planning, cryptography, scheduling, among others, can be directly or indirectly encoded as a SAT problem. Therefore, the development of high-performance SAT solvers has always been a hot research issue.

However, the SAT problem is NP-hard (non-deterministic polynomial time hard) [1], which means that there is no polynomial time algorithm to solve it. Many efforts have been made to design high-performance SAT solvers. It is recognized that solvers based on a complete search technique can achieve good results in solving industrial SAT instances, and solutions based on the stochastic local search technique show effectiveness in solving random SAT instances. The performance of these solvers shows obvious differences in different formula classes. It is speculated that this may be related to the complex structure of a SAT formula [2,3,4,5]. Different conjunctive normal form (CNF) formula classes have different organizational structures, which leads to the differences in performance of SAT solvers. In recent years, researchers have tried to clarify the structures of CNF formulas in order to improve or design high-performance SAT solvers.

Ansótegui et al. studied the community structure of industrial SAT instances from the perspective of complex networks [4,5]. They found that the community structure of industrial SAT instances were obvious while the random SAT instances were not. They verified by experiments that the SAT solvers based on conflict-driven clause learning (CDCL) destroyed the community structure of formulas during a search.

It is useful to study the community structure of CNF formulas. Newsham et al. [6,7] found that the solving time of CDCL solvers was related to the community structure of a SAT instance and used the community structure of the SAT instance to improve the performance of several SAT solvers. Martins [8] used the community structure of formulas to partition soft clauses and then used it to solve the MaxSAT problem. Sonobe et al. [9] improved the performance of a parallel SAT solver by using community structured partitioning. Giráldez-Cru and Levy [10,11] used the formula community to generate some highly modular pseudo-random industrial instances.

Although research on the complex structure of formulas has made some progress, it is still a challenging problem to explain the relationship between the difficulty solving a formula and its structure. In recent years, Li and Pan [12] proposed the structural information theory. They used random walks to capture information interaction between the nodes in graphs and defined the structural entropy of graphs. They defined the structural entropy of a graph to be the minimum overall number of bits required to determine the code of the node that is accessible from a random walk in the graph. In their definition, the structural entropy of graphs is essentially the metric that allows us to fully or maximally detect the K-dimensional structure consisting of the rules, regulations, and orders of the graphs against the random variations occurring in the graphs. It supports the full analysis of networking data and unstructured big data. They used the structural entropy of graphs to study the dynamic evolution of networks, information interaction between nodes, and natural clustering of nodes and made some new progress [13,14,15].

Inspired by structural information theory, in this work, we studied the structural properties of formulas by using the structural entropy of graphs. Combined with the previous research, this paper attempts to give an explanation of the relationship between the difficulty in solving and the complex structure of a formula. The goal is to explore the success rules of SAT solution techniques and to possibly improve them.

The first contribution of this work is to improve an algorithm to solve the two-dimensional structural entropy of graphs. We added a random perturbation to the algorithm E proposed in [12,16] and made it more diversified for the selection of maximum entropy increment. In addition, we also introduced a λ-approximation strategy to approximate the structural entropy of large formulas.

The second contribution of this work is to analyze the relationship between the structural entropy of a formula and the difficulty of its solution and to give an explanation of the difficulty solving the formula from the perspective of structural information theory. First, we represented a CNF formula as a variable graph and then calculated the one-dimensional and two-dimensional structural entropy, community information, and compressing information of the variable graph of the formula. The experimental results showed that the compressing information of a formula is inversely proportional to the difficulty solving the formula. In other words, the smaller the compressing information of a formula, the more complex the essential structure of the formula and, thus, the more difficult the formula is to solve. On the contrary, the bigger the compressing information of a formula, the simpler the essential structure of the formula and the easier it is to solve.

The third contribution of this work is to analyze structural properties of formulas in the process of solving. In particular, We focus on whether the entropy of a variable affects its flipping frequency during solving and focus on the relationship between the selected variables when the local search algorithms encounter a local optimum and the communities to which they belong. The experimental results show the provability of the variable to be assigned or flipped. The variable with large entropy is more likely to be flipped repeatedly. Based on this conclusion, we proposed an initial solution candidate solution generation strategy, which further improved the performance of the solvers CCAsat [17] and Sparrow2011 [18] when integrating this strategy. In addition, the experimental results also showed that a local search solver tends to select variables in different communities for flipping when using it to solve SAT.

The rest of this paper is organized as follows. Some necessary concepts and basic notations are introduced in Section 2. In Section 3, we introduce the methods of graph representation of a formula and provide some definitions about the structural entropy of graphs. In Section 4, we introduce an algorithm to calculate the two-dimensional structural entropy of graphs. An analysis of the structural properties of formulas and the relationship between the structural entropy of a formula and its hardness are presented in Section 5. In Section 6, we present our conclusions.

## 2. Preliminaries

Given a set of Boolean variables $X=\{x_{1},x_{2},\cdots ,x_{n}\}$, a literal refers to the variable *x* or its negation ¬x. A clause *c* of length *s* is a disjunction of *s* literals, i.e., $c=l_{1}\vee l_{2}\vee \cdots \vee l_{s}$; we note s=|c|. We say that a variable x∈c means that *c* contains the literal *x* or ¬x. A CNF formula or a SAT instance of length *t* refers to the conjunction of *t* clauses, $F=c_{1}\wedge c_{2}\wedge \cdots \wedge c_{t}$. A *k*-CNF formula means that the length of each clause in a CNF formula is *k*.

Solving a CNF formula *F* refers to finding a truth assignment τ on a set of boolean variables *X* such that all clauses in *F* are true under τ.

For a CNF formula *F*, we usually use V(F) and C(F) to represent the set of all variables and the set of all clauses that appear in the formula *F*, respectively, and use N(x) to represent the neighboring variable set of the variable *x* in *F*. Two variables *x* and *y* are neighbors if and only if *x* and *y* appear together in at least one clause.

An undirected and weighted graph *G* is an ordered pair G=(V,w), where *V* is a set of vertexes and *w* is a weighted function $w:V\times V\to R^{+}$. For x,y∈V, we have w(x,y)=w(y,x).

**Definition** **1.** *Given an undirected and weighted graph G=(V,w), let N(u) denote the set of neighbor nodes of node u in G. Define the weighted degree of u to be $d_{u}=\sum_{v\in N(u)}w\left(u,v\right)$. For a subset U⊆V, define the volume of U to be $vol\left(U\right)=\sum_{v\in U}d_{v}$. Define $vol\left(G\right)=\sum_{v\in V}d_{v}$ to be the volume of G.*

## 3. Graphical Representation and Structural Entropy of Formulas

### 3.1. Graphical Representation of Formulas

To calculate the structural entropy of a formula, we need to represent a given SAT instance as a graph. Among the existing graph models, the variable graph is a common graph model. In variable graph model, a CNF formula *F* is represented as an undirected and weighted graph G=(V,w). The vertices in *G* represent the variables in *F*, i.e., V=V(F), and the edges in *G* represent the relationship between two neighboring variables. If two variables *x* and *y* are neighbors, we connect them with an edge. *w* is the weighted function of edges in *G*.

**Definition** **2.** *Given a SAT instance F over the set of variables X, the variable graph of F is an undirected and weighted graph G=(X,w), where X is the boolean variables set, and the weight function is as follows:*

(1) wx,y=∑x,y∈c∧c∈F1|c|2.

### 3.2. Structural Entropy of Graphs

Representing a formula as a graph is convenient for us to study the structural characteristics of the formula. Here, we first introduce the definitions of structural entropy of graphs in [12].

**Definition** **3.** *Let G=(V,w) be an undirected and weighted graph with n nodes, and the weight function is w. The one-dimensional structural entropy of G is defined as follows:*

(2) $$\begin{array}{c} H^{1}\left(G\right)=-\sum_{i=1}^{n}\frac{d_{i}}{vol(G)}\log_{2}\frac{d_{i}}{vol(G)}. \end{array}$$

We call the one-dimensional structural entropy of the variable graph of a formula the one-dimensional structural entropy of the formula or the original information of the formula. As we know, entropy is a measure of uncertainty of random variables or random systems. If the entropy of a random variable or random system is larger, then the greater the uncertainty of the variable or the more uncertainty is embedded in the system, the worse the stability of the variable or the system. By calculating the one-dimensional structural entropy of the variable graph of a formula, we can know how much uncertainty is embedded in the formula.

**Definition** **4.** *Let G=(V,w) be an undirected and weighted graph with n nodes, and w is the weight function. Suppose that $P=\{X_{1},X_{2},\cdots ,X_{l}\}$ is a partition of vertices and that P is disjoint; we define the structural entropy of graph G by partition P as follows:*

(3) $$\begin{array}{c} H^{P}\left(G\right)=-\sum_{j=1}^{l}\frac{V_{j}}{vol(G)}\sum_{i\in X_{j}}\frac{d_{i}^{j}}{V_{j}}\log_{2}\frac{d_{i}^{j}}{V_{j}}-\sum_{j=1}^{l}\frac{g_{j}}{vol(G)}\log_{2}\frac{V_{j}}{vol(G)} \end{array}$$

*where $X_{j}$ is called a module or a community, $V_{j}$ is the volume of module $X_{j}$, l is the number of modules, $n_{j}$ is the number of nodes in $X_{j}$, $d_{i}^{j}$ is the weight degree of the ith node of $X_{j}$, and $g_{j}$ is the sum of the weights of the edges with exactly one endpoint in module $X_{j}$.*

**Definition** **5.** *Given a graph G, the two-dimensional structural entropy of G is defined as follows:*

(4) $$\begin{array}{c} H^{2}\left(G\right)=\min_{P}\left\{H^{P}\left(G\right)\right\} \end{array}$$

*where P runs over all the partitions of G.*

The two-dimensional structural entropy of a graph *G* is defined by the partition of vertices in *G*. It contains two parts. The former part, $-\sum_{j=1}^{l}\frac{V_{j}}{vol(G)}\sum_{i\in X_{j}}\frac{d_{i}^{j}}{V_{j}}\log_{2}\frac{d_{i}^{j}}{V_{j}}$, refers to the number of bits needed to determine the code of node *v* in its own module *X*, where *v* is the node accessible from a step of the random walk in G. The second part is $-\sum_{j=1}^{l}\frac{g_{j}}{vol(G)}\log_{2}\frac{V_{j}}{vol(G)}$, which refers to the number of bits needed to determine the code of a module *X*, where *X* is the module accessible from a step of the random walk from nodes outside of *X*.

It can be seen from Definitions 4 and 5 that the two-dimensional structural entropy of a graph is a quantitative measure of the information interaction between nodes in the graph. It measures the minimum amount of information to position the two-dimensional code of the node that is accessible from random walk in *G* with stationary distribution [12]. From the perspective of information theory and coding, the two-dimensional structural entropy of a graph describes the minimum bits required to encode a graph. Therefore, the two-dimensional structural entropy of a graph is understood as an essential structure of the graph and describes how much intrinsic information is hidden in the graph, which cannot be encoded or decoded by any encoder or decoder. By calculating the two-dimensional structural entropy of the variable graph of a formula, we can obtain the essential structure of the formula, i.e., we know how much intrinsic information is hidden in the formula.

It can be seen from the above definition that, for a weighted graph G=(V,w), if there is a partition P of vertices *V* of *G* such that $H^{2}\left(G\right)=\min_{P}\left\{H^{P}\left(G\right)\right\}$, then P is an optimal partition of *V*. We call P a community structure of *G*, in which most of the edges are within a community and few of them connect vertices of distinct communities. We can obtain the modularity of P by partition P. The modularity of P is defined by Newman and Girvan [19] as follows:

(5) $$\begin{array}{c} Q=\sum_{X_{k}\in P}\left[\frac{\sum_{i,j\in X_{k}}w\left(i,j\right)}{vol(G)}-{\left(\frac{\sum_{i\in X_{k}}\sum_{j\in V\;}w\left(i,j\right)}{2vol(G)}\right)}^{2}\right]. \end{array}$$

Note that the modularity defined by Newman and Girvan [19] measures the quality of community structure of a network. They define modularity as the fraction of edges connecting vertices of the same community minus the expected fraction of edges for a random graph with the same number of vertices and same degree. It is different from the definition of the structural entropy of graphs. The structural entropy of graphs is an information theoretical measure of the quality of community structures of graphs. It has many properties, but the definition of modularity has only some of them [16].

In the next section, we present a greedy algorithm to approximate the two-dimensional structural entropy of graphs. By calculating the two-dimensional structural entropy of a graph, we can obtain a partition of vertices of a graph. Here, we continue to present the definition of the compressing information of graphs.

**Definition** **6.** *Given a weighted graph G, we define the compressing information of graph G as $C\left(G\right)=H^{1}\left(G\right)-H^{2}\left(G\right)$. The compression ratio of graph G can be defined as $\rho \left(G\right)=\frac{C(G)}{H^{1}\left(G\right)}$.*

The compressing information of a graph *G* is a quantitative measure, which measures how much uncertainty that can be eliminated is embedded into *G*. Eliminating the amount of uncertainty embedded in *G*, we can obtain the essential structure of *G*. The essential structure of graph *G* is the intrinsic information hidden in *G* that cannot be eliminated by any lossless encoding of *G*.

For a CNF formula *F*, if the two-dimensional structural entropy of the variable graph of *F* is larger, then the essential structure of the formula is more complicated. We have reason to believe that this formula is very difficult to solve. In the following sections, we construct experiments to reveal this problem from the perspective of structural information theory.

## 4. An Algorithm for Calculating Two-Dimensional Structural Entropy

In this section, We describe a greedy algorithm to approximate the two-dimensional structural entropy of graphs.

### 4.1. Algorithm E

First, we introduce the concept of structural entropy increment as follows [16].

**Definition** **7.** *Given a graph G, suppose that $P=\{P_{1},P_{2},\ldots ,P_{l}\}$ is a partition of vertices of G and that $P^{\prime }=\left\{P_{1},P_{2},\ldots ,P_{i-1},P_{i+1},\ldots ,P_{j-1},P_{j+1},{\ldots ,P}_{l},P_{ij}\right\}$ is the new partition after merging modules $P_{i}$, $P_{j}$ in P, where $P_{ij}=P_{i}\cup P_{j}$. We define the structural entropy increment $\Delta_{i,j}^{P}$ as*

(6) $$\begin{array}{c} \Delta_{ij}^{P}\left(G\right)=H^{P}\left(G\right)-H^{P^{\prime }}\left(G\right). \end{array}$$

As can be seen from Definition 7, if $\Delta_{ij}^{P}\left(G\right)>0$, then the partition after merging is better than before. An approximate greedy algorithm E for calculating the two-dimensional structural entropy of graphs is described as follows:

(1) First, each node *v* of graph *G* is divided into a single set. That is, if $P=\{P_{1},P_{2},\cdots ,P_{n}\}$ is the initial partition, then $P_{i}=\left\{v_{i}\right\}$.
(2) For any 1≤i,j≤n, compute $\Delta_{ij}^{P}\left(G\right)$.
(3) If there is no 1≤i,j≤n, such that $\Delta_{ij}^{P}\left(G\right)>0$, then terminate and output the partition P. Otherwise, find i,j such that $\Delta_{ij}^{P}\left(G\right)$ is maximized among all $\Delta_{ij}^{P}\left(G\right)$, then merge $P_{i},P_{j}$. Let $P_{ij}=P_{i}\cup P_{j}$, $P=\{P_{1},P_{2},\ldots ,P_{i-1},P_{i+1},\ldots ,P_{j-1},P_{j+1},{\ldots ,P}_{l},P_{ij}\}$, and go back to (2).
(4) Compute the two-dimensional structural entropy of graphs by P according to Equation (3).

The time complexity of algorithm E is $O(n\log^{O(1)}n)$, which means that we can quickly calculate the two-dimensional structural entropy of a formula with a certain scale.

In algorithm E, each iteration always selects the modules with the largest $\Delta_{ij}^{P}\left(G\right)$ to merge. We think such conditions are too strict. In practice, we found that merging slightly smaller $\Delta_{ij}^{P}\left(G\right)$ produces better results. Therefore, we add a random perturbation to the algorithm E, and step (3) is modified as follows:

(3) If there is no 1≤i,j≤n, such that $\Delta_{ij}^{P}\left(G\right)>0$, then terminate and output the partition P. Otherwise, Among the first *q* largest $\Delta_{ij}^{P}\left(G\right)$, randomly select a $\Delta_{ij}^{P}\left(G\right)$, find the corresponding i,j, and merge $P_{i},P_{j}$ to obtain the partition P. Finally go back to (2).

Note that there are some restrictions on the first *q* largest $\Delta_{ij}^{P}\left(G\right)$. Small increments of $\Delta_{ij}^{P}\left(G\right)$ are useless to us. Therefore, the difference between the values of the first *q* largest $\Delta_{ij}^{P}\left(G\right)$ should be small and the variance should be as small as possible. In our experiment, the value of *q* is set as follows: (i) Select the first *s* structural entropy increments $\Delta_{ij}^{P}\left(G\right)$ in turn, and calculate the average structural entropy increment $\Delta_{mean}$. (ii) If the maximum entropy increment $\Delta_{max}\cdot 0.99<\Delta_{mean}$ and s≤10, then set q=s; otherwise, set q=10.

### 4.2. λ-Approximation Strategy

In addition, in order to quickly calculate the two-dimensional structural entropy of some large instances, we propose a λ-approximation strategy. This strategy is based on the singular value decomposition (SVD) of matrices. In other words, an m×n real matrix *A* can be factored into $A=U\Sigma V^{T}$, where *U* and *V* are unit orthogonal matrices, Σ is a diagonal matrix, and the elements on the main diagonal are the eigenvalues.

Given a CNF formula *F* with variables set *X* and clauses set *C*, our λ-approximation strategy algorithm is described as follows:

(1) Construct the formula matrix $M_{n\times m}$. Here, *n* is the number of variables and *m* is the number of clauses in *F*. If the variable *x* appears in clause *c*, then set $M_{x,c}=1$ (positive occurrence) or $M_{x,c}=-1$ (negative occurrence). Otherwise, set $M_{x,c}=0$.
(2) Compute the singular value of matrix *M*.
(3) Select the first λ eigenvalues in Σ to reconstruct the formula matrix $M_{n\times m}^{*}$, where $M_{n\times m}^{*}=U_{n\times \lambda }\Sigma_{\lambda \times \lambda }^{*}V_{\lambda \times m}^{T}$. Considering the matrix $M_{n\times m}^{*}$ as the adjacency matrix of a graph, we can calculate the one-dimensional and two-dimensional structural entropies of the graph by $M_{n\times m}^{*}$.

In the next subsection, we carry out experimental studies to illustrate the value of the parameter λ and verify the effectiveness of the strategy.

### 4.3. Experimental Evaluation

**Figure 1:**
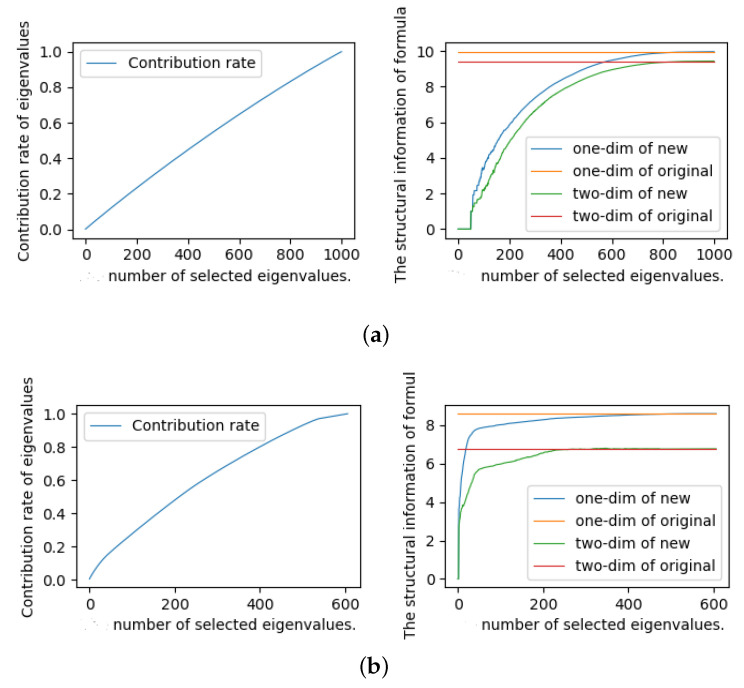
The contribution rate of eigenvalues and the approximate situation of structural information for instances A and B. The X-axis represents the number of selected eigenvalues in formula matrix, where the eigenvalues have been arranged from largest to smallest. (**a**) Random instance A (contains 1000 variables); (**b**) Industrial instance B (contains 600 variables).

First, we designed some experiments to study the value of λ. We generated random 3-SAT instances (10 instances in each group) with different numbers of variables (n=20,50,100,500,1000) and different numbers of clauses (m=91,218,403,2130,20,000) for the experiments. At the same time, we also selected some instances from the SAT competition 2018 industrial benchmark for experiments. In the experiment, we calculated the eigenvalues of each formula matrix and sorted the eigenvalues from large to small. Then, we calculated the cumulative contribution rate of eigenvalues and the one-dimensional and two-dimensional structural entropy of formulas under the contribution rate. Some experimental results are shown in Figure 1. The experimental results of other instances are basically consistent with Figure 1. We will not show them here.

As we can seen from Figure 1, we found that the structural entropy of the new formula can effectively approximate the structural entropy of the original formula when the cumulative contribution rate of selected eigenvalues exceeds 70% whether it is a random formula or an industrial formula. Therefore, in the subsequent experiments, we set the value of λ as the number of selected eigenvalues, where the cumulative contribution rate of selected eigenvalues was not less than 70%.

**Figure 2:**
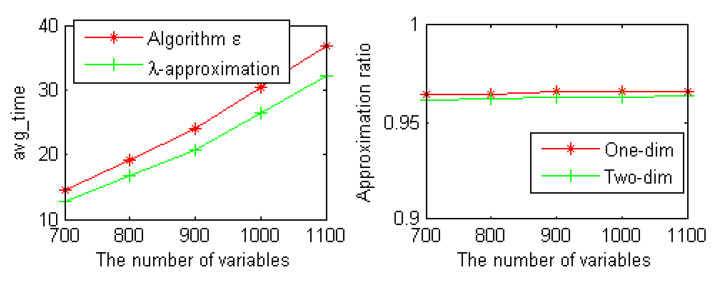
The average running time of two algorithms and the approximation ration.

Second, we carried out experimental studies to evaluate the effectiveness of the λ-approximation strategy. We calculated the structural entropy and the running time of each group of instances on large random 5-SAT instances from the SAT competition 2009 benchmark by adopting the algorithm E and the λ-approximation strategy. These instances were relatively complex and larger than the random 3-SAT instances used in Figure 1. The experimental results are shown in Figure 2.

As can be seen from Figure 2, the average running time of our λ-approximation strategy is better than algorithm E on random 5-SAT instances. Compared with the original formula, the approximate ratio of one-dimensional and two-dimensional structural entropy of the new formula is more than 96%.

**Figure 3:**
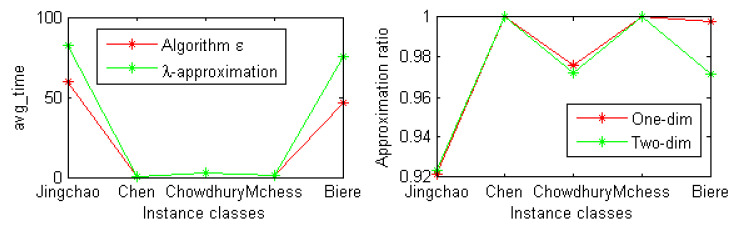
The average running time of two algorithms and the approximation ration.

At the same time, we also conducted experiments on some instances of the SAT competition 2018 industrial benchmark. The experimental results are shown in Figure 3. From Figure 3, we can see that the average running time of our λ-approximation strategy is slightly worse than that of algorithm E on classes *Jingchao* and *Biere*. Compared with the original formula, the approximate ratio of one-dimensional and two-dimensional structural entropies of the new formula is more than 92%.

## 5. Structural Characteristics of Formulas

The structure of a CNF formula is very complex, and the variables are related to each other by some clauses. In fact, the variable graph model we introduced is a good description of this complex structure. In general, a variable with more occurrences in a formula is subject to more constraints, and it should be connected with more edges. Our variable graph model just satisfies this condition. Therefore, analyzing the structural entropy of the variable graph of a formula may help us further understand the complex structure of the formula.

### 5.1. Structural Entropy of Formulas

To understand the complex structure of formulas, we carried out experiments to analyze the structural entropy of formulas. Our first experiment studied the structural entropy of random 3-SAT instances. We generated a number of random 3-SAT instances (10 instances in each group) with different clause to variable ratios α=m/n=1,2,⋯,7, for a fixed number of variables $n=10^{4}$. Simultaneously, we also generated a certain number of random 3-SAT instances (10 instances in each group) with a fixed constraint ratio α=4.26 and different number of variables $n=10^{2},10^{3},10^{4},10^{5}$. Then, we reported the modularity *Q* of the partition returned by our algorithm as well as the one-dimensional one-dim and two-dimensional two-dim structural entropies of these formulas. We also reported the compressing information C and compression ratio ρ. Table 1 and Table 2 show the results.

As we can see, the modularity *Q* and the compression ratio ρ of random 3-SAT instances with a fixed number of variables are very low (see Table 1). As the constraint ratio increases, the two-dimensional structural entropy of random 3-SAT instances gradually increases, which is very close to the one-dimensional structural entropy. We can also see that the compressing information C of these formulas decreases with the increase in the constraint ratio α. For random 3-SAT instances with a constraint ratio near the phase transition point (see Table 2), the modularity *Q* and compression ratio ρ did not change much with the increase in the number of variables *n* and their values are very low. The two-dimensional structural entropy of these formulas are very close to the one-dimensional structural entropy, and the compression ratio is less than 15%.

For random 3-SAT instances, the difficulty solving them gradually increases with the increase in the constraint ratio α and it is very difficult to solve when the constraint ratio is close to the phase transition point [20,21,22]. From the experiment, we observe that, as the constraint ratio increases, the two-dimensional structural entropy of this formulas gradually increases to be close to the one-dimensional structural entropy and the compressing information of formulas gradually decrease to less than 15% of the original information. We can conclude that the difficulty solving a formula is related to the compressing information of the formula. If the two-dimensional structural entropy of a formula is close to the one-dimensional structural entropy, that is, the compressing information of the formula is very little, then the intrinsic information hidden in the formula is bigger and the essential structure of the formula is more complex due to the large amount of uncertainty embedded in the formula, which makes the formula more difficult to solve.

In addition, we also observe that the community structure of random 3-SAT instances is not obvious except the constraint ratio α=1. This finding is consistent with the finding in [4,5]. Note that a network is considered to have a distinct community structure when the module value of the network is in the interval [0.3,0.7] [19].

In the second experiment, we analyzed the structural entropy of industrial SAT instances. As far as we know, it is relatively easy to solve industrial SAT instances. Now, we observe the structural entropy of industrial SAT instances by experiments and see if the compressing information of industrial SAT instances is relatively large. Our experiment was operated on the industrial benchmark from SAT competition 2018. We also report the one-dimensional and two-dimensional structural entropies, community structure, and compressing information of each instance class. The results are shown in Table 3.

In Table 3, the notations instance_class and #ins represent instance classes and the number of instances in each instance class, respectively. As you can see, the community structure of the industrial SAT instance is very obvious and it is consistent with the finding in [4,5]. The gap between two-dimensional structural entropy and one-dimensional structural entropy of industrial SAT instances is obvious, that is, the compressing information of industrial SAT instances is large. Meanwhile, we can also see that the compression ratio of industrial SAT instances is generally higher than 15%. This shows that a large amount of uncertainty embedded in the formula can be eliminated and that the essential structure of the formula is relatively simple. This may be one reason why industrial SAT instances are relatively easy to solve.

Through experiments, we can conclude that the structural entropy of a formula can approximately measure the complex structure of the formula. For a given CNF formula *F*, if the two-dimensional structural entropy of formula *F* is larger, then the essential structure of *F* is more complex. At the same time, we can see that the compressing information of a formula can approximately measure the difficulty solving the formula. If the compression information of a formula is larger, then a large amount of uncertainty embedded in formula can be eliminated, the essential structure of the formula is relatively simple, and the formula will be easier to solve. On the contrary, if the compressing information of a formula is smaller, then the uncertainty that can be eliminated in the formula is less, the essential structure of the formula is more complex, and the formula is more difficult to solve.

Now, a natural question is as follows: since the structural entropy of a formula can approximately measure the complex structure of the formula, does the structural entropy of the formula contribute to solving the formula? We continued to construct experiments to reveal this problem.

### 5.2. Structural Properties of Formulas during Solving

Given a formula *F*, *G* is the variable graph of *F*. The entropy of node *u* in *G* is defined as $H\left(u\right)=-\frac{d_{u}}{vol(G)}log_{2}\frac{d_{u}}{vol(G)}$, which represents the uncertainty of random walks in *G* arriving at node *u*. The one-dimensional structural entropy of *G* is the sum of these uncertainties. Entropy describes the uncertainty of a random variable or a random system. In a variable graph, the greater the entropy of a node, the greater the degree of uncertainty freedom of the node. Therefore, we conjecture that the probability of the variable (node) with a large entropy to be flipped is high when solving the formula with a local search solver.

**Figure 4:**
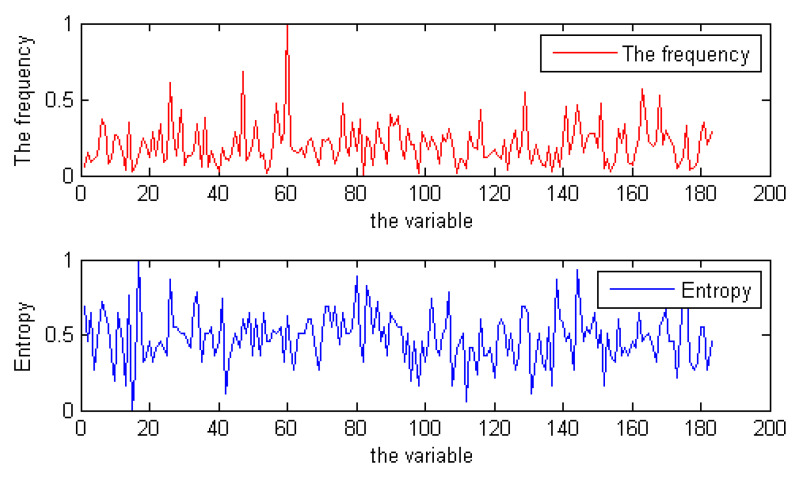
The flip times and entropy of variables (only some variables are shown) for the instance unif-k3-r4.2-v2000-c8400-S1494472801-040 during a search. These data have been normalized.

To verify our conjecture, we used the local search solver CCAsat to solve these random instances that were used in SAT competition 2009. At the same time, we also used the local search solver CCAsat to solve some industrial instances from SAT competition 2018. CCAsat is a competitive local search solver for random SAT instances, which was proposed by Cai in [17]. In the process of solving, we separately counted the number of times each variable was flipped and computed its entropy. Figure 4 shows the relationship between the frequency of a variable being flipped and its entropy for random instance unif-k3-r4.2-v2000-c8400-S1494472801-040 in the process of solving. Due to limited space, we do not show the statistical results for other instances, including industrial instances, but their results are highly consistent with the results shown in Figure 4.

From the experiment, we can observe that, indeed, the greater the entropy of a variable, the greater the frequency of the variable being flipped. This conclusion is useful to us. On the one hand, it can further explain our previous conclusion, i.e., the difficulty solving a formula is related to the structural entropy of the formula. If there are many variables with large entropy in a formula, it will take more time to fix the values of these variables when solving the formula. Therefore, it is difficult to solve. On the other hand, we can use it to improve some local search solvers for SAT. For a given formula *F*, if the structural entropy of a variable is large, which means that the probability of this variable being repeatedly assigned is higher when solving *F* with a local search solver, then we can generate a better initial candidate solution according to the structural entropy of variables when solving *F* and use it to guide the algorithm to search.

Given a formula *F*, *G* is the variable graph of *F*. The process of generating an initial candidate solution with the structural entropy of variables is as follows.

(1) Calculate the structural entropy of nodes in *G*.
(2) Reorder variables according to the structural entropy (from small to large).
(3) Take out the sorted variables in turn and count their positive and negative occurrence times in the remaining unsatisfied clauses. If the times of positive occurrence are more than negative, then assign the value of the variable to 1 and to 0 otherwise.

**Figure 5:**
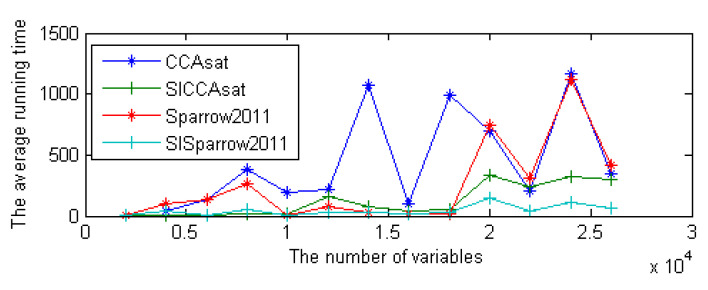
The performance comparison results of the solvers CCAsat, Sparrow2011, SICCAsat, and SISparrow2011 on the SAT competition 2009 benchmark.

This strategy can be effectively integrated into some other local search solvers for SAT. In our previous work, we integrated this method and clause weighting strategy into the solvers CCAsat and Sparrow2011 [18], resulting in solvers SICCAsat and SISparrow2011, which further improved their performance. Figure 5 shows the performance of these solvers on the SAT competition 2009 benchmark.

**Figure 6:**
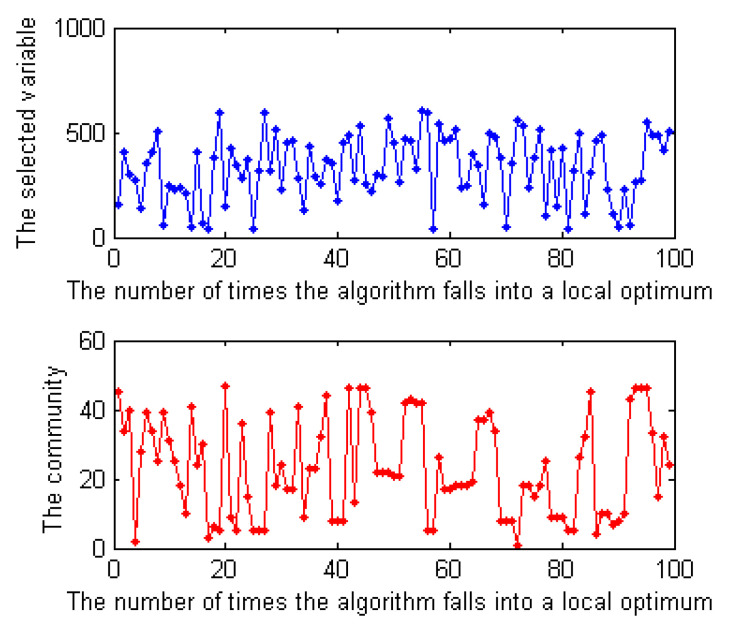
The selected variables and the communities to which they belong for the instance CBS_k3_n100_m403_b10_55 during a search. The horizontal axis represents the number of times that the algorithm encounters a local optimum when solving the instance with the local search solver SICCAsat, and the vertical axis represents the selected variables and the communities they belong to.

We designed experiments to analyze the structure of a formula during a search. Ansótegui et al. [5] have verified by experiments that the community structure of industrial SAT instances is gradually destroyed with the increase in iteration times when using a complete solver CDCL to solve it. Here, we solved a SAT instance with the local search solver SICCAsat and observed whether the instance shows some structural characteristics during a search. In the experiment, we outputted each flipped variable and the community it belongs to when the solver encounters a local optimum. Figure 6 shows the results of the solver on a random instance. The numerical results are shown in Table 4. In Table 4, the notation times represents the number of times a algorithm encounters a local optimum, var represents the selected variables, and com represents the communities to which the selected variables belong. Since the results of other SAT instances are consistent with the one showed in Figure 6, we do not show more results here.

As shown in Figure 6, to jump out of the local optimum, the variables selected by the solver each time are different in most cases and the communities to which they belong are also different. This shows that a local search solver tends to select variables in different communities to flip on each iteration.

Combining the research of Ansótegui et al. [5], we can conclude that, when solving SAT instances with a solver, the solver tends to select variables among different communities to form a new learning clause (CDCL) or perform the next round of searches (local searches). This conclusion may be useful to us. After understanding the structural characteristics of a formula in the process of solving, we may be able to develop some high-performance SAT solvers.

## 6. Conclusions

Modern SAT solvers show obvious differences in solving different formula classes. The reason may be that different SAT formula classes have different structural characteristics. In this work, we studied the structural properties of CNF formulas and discussed the relationship between the structural properties of a formula and its solving difficulty.

Firstly, we represented a CNF formula as a graph, on which we studied the structural entropy and community structure of the formula. In the experiment, we found that the compressing information of a formula decreases with an increase in the constraint ratio for random 3-SAT instances. When the constraint ratio is close to the phase transition point, the compressing information of the formula gradually decreases to less than 15% of the original information. For industrial SAT instances, the compressing information is generally larger than 15% of the original information, and the gap between two-dimensional structural entropy and one-dimensional structural entropy is larger than random SAT instances. Therefore, we believe that the difficulty solving a formula is related to its structural entropy. Meanwhile, we gave an explanation of the difficulty solving a formula from the perspective of information theory. In addition, we also found that the community structure of industrial SAT instances are obvious while the random SAT instances are not.

Secondly, we proposed a λ-approximate strategy to approximate the two-dimensional structural entropy of formulas. The strategy is based on singular value decomposition of a matrix. The experimental results verified the effectiveness of the strategy on large random SAT instances.

Finally, we analyzed the structural properties of formulas in the process of solving. In particular, we experimentally verified that the structural entropy of a variable affects the frequency of the variable being flipped. By using this conclusion, we proposed an initial solution generation strategy for some stochastic local search solvers. Combined with the clause weight method, this strategy can effectively improve the performance of some local search solvers. In addition, we experimentally verified that, in solving SAT instances with a solver, the solver tends to select variables in different communities to form new learning clauses (complete solvers) or perform the next round of searches (local search solvers).

The study of the complex structure of formulas has significant implications for better understanding why some solvers perform better on industrial SAT instances and why others perform better on random SAT instances. Moreover, we can use these findings to develop or improve some existing solvers for SAT. Meanwhile, this analysis also serves as the basis for new random SAT generation models that produce more realistic pseudo-industrial random instances. This problem is distinguished as one of the ten challenge problems in SAT [23,24,25].

The findings in the present paper not only help us to design new SAT solvers but also help us to construct harder instances by the principle of structural entropy of CNF formulas.

## Figures and Tables

**Table 1:** The structural information of random 3-SAT instances with different α and $n=10^{4}$.

*n*	α=m/n	*Q*	ρ	One-Dim	Two-Dim	C
10,000	1	0.419	0.321	13.027	8.841	4.186
10,000	2	0.228	0.172	13.162	10.901	2.261
10,000	3	0.161	0.118	13.206	11.652	1.554
10,000	4	0.130	0.090	13.227	12.033	1.194
10,000	4.25	0.125	0.085	13.231	12.103	1.128
10,000	4.5	0.121	0.081	13.234	12.161	1.072
10,000	5	0.113	0.074	13.239	12.260	0.979
10,000	6	0.101	0.064	13.247	12.402	0.845
10,000	7	0.091	0.057	13.253	12.498	0.756

**Table 2:** The structural information of random 3-SAT instances with different numbers of variables and fixed clause to variable ratios α.

*n*	α=m/n	*Q*	ρ	One-Dim	Two-Dim	C
100	4.26	0.164	0.132	6.587	5.718	0.868
1000	4.26	0.147	0.098	9.909	8.936	0.974
10,000	4.26	0.125	0.085	13.231	12.104	1.126
100,000	4.26	0.111	0.085	16.552	15.138	1.414

**Table 3:** The structural information of industrial SAT instances.

Instance_Class	#ins	*Q*	ρ	One-Dim	Two-Dim	C
biere	20	0.650	0.490	13.031	6.646	6.385
chen	20	0.159	0.103	8.319	7.464	0.855
chowdhury	19	0.534	0.332	9.249	6.179	3.069
deriendt	15	0.918	0.332	11.585	7.739	3.846
harder	11	0.803	0.366	15.930	10.093	5.837
heule	20	0.694	0.261	14.393	10.640	3.754
Heusser	17	0.600	0.413	16.737	9.827	6.910
jingchao	20	0.366	0.223	9.752	7.578	2.173
scheel	20	0.674	0.497	14.817	7.457	7.360
Porkhunov	9	0.670	0.423	10.674	6.159	4.514

**Table 4:** The selected variables and the communities they belong to for the instance CBS_k3_n100_m403_b10_55 during a search.

Times	var	com	Times	var	com	Times	var	com	Times	var	com	Times	var	com
1	156	45	21	422	9	41	450	8	61	515	18	81	44	5
2	402	34	22	346	5	42	491	46	62	237	18	82	318	5
3	301	40	23	283	36	43	272	13	63	250	18	83	498	26
4	274	2	24	369	15	44	533	46	64	398	19	84	114	32
5	141	28	25	44	5	45	255	46	65	345	37	85	309	45
6	354	39	26	318	5	46	222	39	66	159	37	86	456	4
7	402	34	27	596	5	47	300	22	67	500	39	87	484	10
8	502	25	28	313	39	48	286	22	68	475	34	88	227	10
9	57	39	29	516	18	49	564	22	69	377	8	89	115	7
10	245	31	30	225	24	50	454	21	70	51	8	90	51	8
11	224	25	31	453	17	51	262	21	71	350	8	91	227	10
12	238	18	32	458	17	52	469	42	72	562	1	92	55	43
13	206	10	33	281	41	53	463	43	73	528	18	93	263	46
14	49	41	34	132	9	54	327	42	74	238	18	94	269	46
15	410	24	35	429	23	55	605	42	75	380	15	95	547	46
16	63	30	36	288	23	56	596	5	76	516	18	96	488	33
17	40	3	37	253	32	57	44	5	77	100	25	97	483	15
18	378	6	38	368	44	58	544	26	78	411	9	98	415	32
19	596	5	39	350	8	59	458	17	79	143	9	99	503	24
20	148	47	40	172	8	60	466	17	80	422	9	100	199	24

## Data Availability

Some data benchmarks are available from http://satcompetition.org/ (accessed on 8 January 2021).
